# Hotspots of MLV integration in the hematopoietic tumor genome

**DOI:** 10.1038/onc.2016.285

**Published:** 2016-11-07

**Authors:** T Tsuruyama, T Hiratsuka, N Yamada

**Affiliations:** 1 Department of Pathology, Kyoto University, Graduate School of Medicine, Sakyo-ku, Kyoto, Japan; 2 Center for Anatomical, Pathologic and Forensic Medical Research, Graduate School of Medicine, Kyoto University, Kyoto, Japan

## Abstract

Extensive research has been performed regarding the integration sites of murine leukemia retrovirus (MLV) for the identification of proto-oncogenes. To date, the overlap of mutations within specific oligonucleotides across different tumor genomes has been regarded as a rare event; however, a recent study of MLV integration into the oncogene *Zfp521* suggested the existence of a hotspot oligonucleotide for MLV integration. In the current review, we discuss the hotspots of MLV integration into several genes: *c-Myc*, *Stat5a* and *N-myc*, as well as *ZFP521*, as examined in tumor genomes. From this, MLV integration convergence within specific oligonucleotides is not necessarily a rare event. This short review aims to promote re-consideration of MLV integration within the tumor genome, which involves both well-known and potentially newly identified and novel mechanisms and specifications.

## Introduction

Retroviruses such as avian leukosis retrovirus,^1^ murine leukemia retrovirus (MLV)^2^ and feline leukemic retrovirus^3^ are involved in the development of a wide variety of diseases including malignant neoplasms, most particularly soft tissue tumors or hematopoietic tumors including lymphoma and leukemia. Retroviral genomes are integrated into the host cellular genome, becoming proviral genomes that contribute to tumorigenesis; this step is one of the essential steps in the retroviral life cycle. The inserted retroviral genome (the provirus) is thus replicated stably in the course of host cell division.^4^ By becoming a part of host genome, the provirus promoter and enhancer elements can instead promote downstream gene expression.^2^ When the provirus is inserted into the vicinity of cellular oncogenes controlling host cell growth and expansion, the affected cells will occupy the lymph node tissues and form lymphoma tissues.^5, 6^ To date, many researchers have analyzed the retrovirus integration sites, in order to identify neighboring oncogenes that are transcriptionally activated in the tumor genome.^5^

MLV integration represents an old but still relevant unsolved problem in the field of oncology. To identify the selectivity of genomic virus integration sites, the vast majority of previously reported studies have used methods involving the infection of cell culture systems with retroviruses, followed by the subsequent identification and comparison of respective integration sites in the transfected cell genomes.^7^ Previous studies have shown that γ-retroviruses, including MLV, exhibit strong bias for integrating near active gene promoters and associated CpG islands.^8, 9, 10, 11^ On the other hand, there are reports that transcriptionally active genes are favored by integration,^8^ or that enhancers are major target of MLV vector integration.^10^ As discussed later in this review, the bromodomain and extraterminal domain (BET) family of proteins are noted as the key factor of MLV integration near transcription start sites.^12^ In particular, whole genome analysis using second-generation sequencing has facilitated the recent discovery that MLV integration site selection is driven by strong enhancers and active promoters in an integration assay using cultured cells.^9, 10^ DNA secondary structural fluctuation around an integration hotspot might contribute to the open chromatin structure in the transcriptionally active site that would likely promote proviral integration after the physical breakage of the double-stranded DNA (dsDNA).^13, 14^

In association with MLV integration bias, there have been extensive discussions regarding the clinical application of MLV vectors. For gene therapy, an MLV-based vector was found to have integrated into the *LIM domain only 2* (LMO2) gene in human T cells in a gene therapy trial^15, 16^ and thereby induced T-cell leukemia in the two patients.^17, 18, 19, 20^ However, the majority opinion was that the selection of *LMO2* as an insertion site in the patients was accidental. In addition, the tumorigenesis caused by MLV insertion has become an issue of concern with respect to the clinical application of induced pluripotent stem (iPS) cells, whereas the present protocol for iPS cell preparation uses a plasmid vector that does not have tumorigenesis potential.^21^

## A model mouse strain of MLV-induced lymphomagenesis

As an experimental model for studies of MLV-induced tumorigenesis, an inbred strain of mice such as AKR can be used that is susceptible to spontaneous tumors by the integration of MLV;^22^ this susceptibility arises from endogenous (that is, previously genetically acquired in the germ line) MLV provirus genomes.^23^ BXH2^24^ and AKXD^25^ are well-known mouse strains that have been used to study the process of leukemogenesis. Using these strains and other strains, the identification of integration sites has been a powerful tool for the study of oncogenes. In fact, many integration sites have been registered in the retroviral-tagged cancer gene database (http://variation.osu.edu/rtcgd/), which has in turn been used for the study of retroviral tumorigenesis, wherein a numbering system based on the ecotropic viral integration site (*EviN*; *N*=1, 2, 3,..) was applied by Copeland and colleagues,^26, 27^ to summarize the MLV integration site. Notably, many integration sites within identical genes are registered in the database; however, integration site overlap at the oligonucleotide level is rare in this database with the exception of *c-Myc*.

## SL/Kh strain

SL/Kh represents an inbred mouse strain derived from AKR that we have developed previously and wherein we have achieved MLV-mediated induction of lymphoma.^28, 29, 30, 31^ One of the advantages of using this strain for the study of integration is the extremely biased integration into specified genes that occurs in these mice, as observed in tumors. This strain possesses pathogenic endogenous ecotropic murine virus 11 (*Emv11*) that has been mapped onto chromosome 7 and is shared with the AKR mouse strain.^23, 32^ In the SL/Kh strain, the pro- to pre-B cells in the bone marrow expand^28, 33^ and newly acquire more than one copy of the proviral genome during the process of lymphomagenesis;^34^ >90% of these mice spontaneously develop sIgM- pro- or pre-B lymphomas that are positive for BP-1 by the age of 6 months.^28, 32^ Notably, the phenotypic stability and the tumor incidence are significantly higher than those in other models, BXH2^24^ and AKXD.^25^ We have thus used the SL/Kh mouse strain to investigate MLV integration because of the high incidence and reproducibility of the phenotype.^29^

## Phenotypes of MLV-induced lymphomagenesis

In the reported cases of spontaneous hematopoietic tumors, granulocytes, B cells, T cells and their immature lineages serve as host cells and can develop tumors such as leukemia or lymphoma following MLV infection. The tumor phenotype in tumor-susceptible strains might be attributed to the host genetic background of the mice,^22, 24, 35^ as well as viral tropism and the function of target genes.^36^ Tropism of the viruses, that is, which type of cells are infected by viruses, is determined by the presence of the retroviral receptor on the host cells and the Env protein on the viral particle;^37^ both myeloid and lymphoid cell lineages express retroviral receptor. In addition, the capsid domain of Gag polyprotein, which is the major structural protein among the MLV-encoded proteins, mediates the assembly of many retroviral proteins. Capsid confers tropism that determines the susceptibility to the intracellular restriction factors Friend virus susceptibility1 (*Fv1*) on chromosome 4 that inhibits the development of MLV by interaction with capsid.^38^ Tumor phenotype might therefore directly depend on the gene structure of proviral genome. Moloney MLV induces almost exclusively lymphoid disease; however, when replacing the long terminal repeat^39^ with that of retrovirus 4070A, the MLV causes myeloid leukemias.^39^

With respect to the genetic background of mice, we previously identified a locus responsible for pre-B-cell expansion, *Bomb1* near Mit319 on chromosome 3, via the genetic study of SL/Kh. A congenic mouse on a NFS/N mouse background carrying the *Bomb1* locus of SL/Kh mice demonstrated expansion of pre-B-cell lymphocytes^28, 33^ and microsatellite instability near *Bank1* gene locus, an adaptor molecule of pre-B receptors in pre-B-cell lymphomas in SL/Kh mice.^40^ Therefore, the microsatellite instability of the responsible locus as well as other background factors might cooperatively determine the phenotype of the tumor.

## Retroviral genome integration into the host genome

### Molecular integration mechanism

The molecular biology of MLV integration is fairly well understood. Following retroviral infection via viral receptor molecules on host cell membrane, viral dsDNA synthesis by reverse transcriptase begins in the cytoplasm and is completed before or after virus entry into the nucleus. A viral enzyme integrase (IN) that is encoded in the retroviral genome cleaves the termini of the viral DNA; the viral dsDNA then forms a pre-integration complex and enters the nucleus. IN forms a tetramer or oligomer at the terminus of the pre-integration complex DNA; these catalyze the host DNA cleavage reaction in a staggered manner. It has been suggested that the terminal ends of the dsDNA might be critical for integration, and that the presence of a dinucleotide capsid at the terminal 3′-ends is particularly essential.^13, 41^ Subsequently, the free energy change released from the broken phosphodiester bonds in the host dsDNA promotes the formation of new bonds joining the viral DNA ends to the ends of cleaved host DNA. DNA synthesis extends from the host DNA flanking the host-viral DNA junctions and fills in the gaps adjacent to the viral DNA, displacing the viral DNA ends. Consequently, four bases are duplicated on either side of the MLV proviral genome; during HIV-1 integration, five nucleotides are duplicated.^42^ These duplicated nucleotides have been analyzed from the perspective of integration selectivity; however, no significant tendency of the sequence motifs has been identified. For this reason, integration has been believed to be random as for the target nucleotides.

## Hotspots of MLV integration

Using the tumor DNAs from spontaneous tumor model mice such as BXH2, AKXD and SL/Kh, inverse PCR was performed,^26, 42, 43^ which involves digestion of the host cell DNA with a restriction enzyme such as *Sac*II, which recognizes CpG islands near gene-coding regions, followed by a ligation reaction using T4-ligase.^36^ The development of inverse PCR-dependent cloning has allowed the identification of numerous integration sites that have in turn been used for the identification of novel oncogenes^26^ such as *Stat5a*,^43^ *Hipk2*, *Fiz1*^44^ and *Zfp521*,^45^ which are frequent integration loci. Before this technical development, the distribution of integration sites within a given gene were not sufficiently investigated and there were few studies providing detailed data regarding the integration within a short segment in a single gene. Here, the term ‘hotspot of integration' is used to imply that the integration site is shared by more than one different tumor, despite the fact that the probability is prospected to be substantially lower for integration into such a narrow range.

### Hotspots in *c-Myc* and *N-myc*

**Figure 1:**
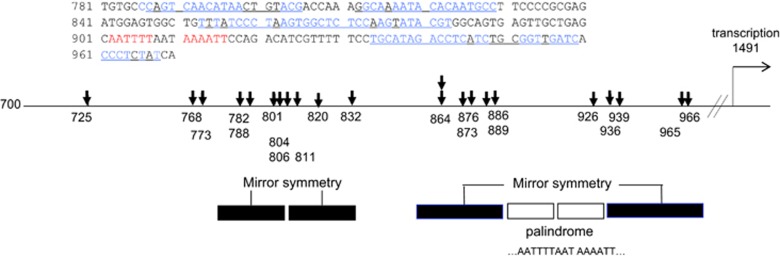
MLV integration sites within *c-myc* (M12345). Sequence motifs in the *c-myc* promoter. The shown sequence represents the mirror symmetry in blue and palindrome in red. The white and black boxes with lines represent the pairs of palindromic and mirror symmetry motifs, respectively. The downward arrows indicate the previously reported integration sites shown by the numbers.

Systematic analysis of integration site has been limited to the study of integration into the *c-myc* proto-oncogene.^46, 47, 48^ Here we reviewed MLV integration sites within the *c-myc* promoter area in particular. As mentioned previously, the systematic analysis did not elucidate a definite trend. However, several integration sites were shared by tumors in various strains of mice, suggesting that certain sites might be preferred by MLV integration.^49^ In a systematic analysis of SL3-3 MLV integration sites, many nucleotides were shared by different tumors. For instance, nucleotide numbers 773, 832, 864 and 889 (M1234.5 in GENBANK) were actually shared by different tumors in different strains (Figure 1 and Table 1). We noted that *c-Myc* promoter consists of palindromic and mirror symmetry sequence motifs around the integration sites (Figure 1).

**Figure 2:**
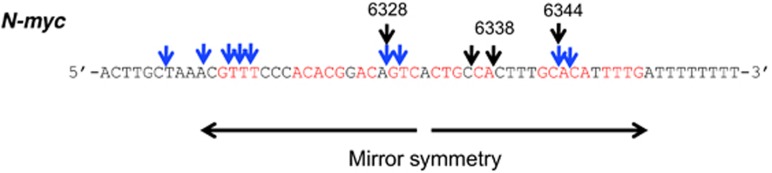
MLV integration within *N-myc* (number 6300-, M12731) (Table 1). The red letters indicate the mirror symmetry motif in the *N-myc* gene. The two vertical arrows indicate the paired regions of mirror symmetry. The black and blue downward arrows represent the integration sites in SL/Kh mice and other previously reported sites.

Similarly, MLV integration sites were shared by lymphoid tumors in the third exon and the 3′-flanking sequence in *N-myc*.^50, 51^ Numbers 6328 and 6344 (M12731) were shared by different tumors in different strains and mirror symmetry sequence motifs around the integration sites (Figure 2).

### Hotspots in *Stat5a*

Next, we consider the MLV integration sites in *Stat5a.* The encoded STAT5A protein is a member of the signal inducer and activator of transcription (STAT) family. These proteins form a dimer that translocates into the nucleus and exerts transcriptional activity by binding to the γ-interferon activation site palindromic element in the promoters of target anti-apoptotic genes.^52, 53^ The products of these genes, such as *c-Myc*, *Pim-1*, *Bcl-xL* and *Cyclin D1*,^54^ regulate proliferation and anti-apoptosis in hematopoietic cells; STAT5A in particular contributes to IL-7-induced B-cell precursor expansion.^43, 49^

**Figure 3:**
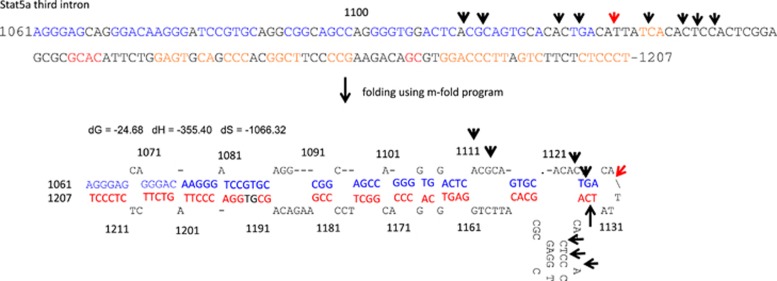
MLV integration sites within *Stat5a.* The sequence represents the whole sequence of AF049104 from (5′-)1061th–1207th nt (-3′) (*Stat5a* gene sequence in GenBank, renumbered according to the nucleotide number form the start of the gene, BLAST: https://blast.ncbi.nlm.nih.gov). The colored letters in blue and red represent the pair of palindromic sequences in the above sequence of which stem of the hairpin structure that is anticipated by *m-fold* analysis. dG, dH and dS represent Gibbs free energy, enthalpy, and entropy change (kcal/mol) in folding. [Na+]=1.0 mM. Folding temperature is equal to 37°C. The most frequent site (N0.1130, shown by a red arrow) that is shared by different pre-B-cell tumor is shown.^41^

In pre-B-cell lymphoma in SL/Kh mice, MLV integration within the 400 bp second intron of the *Stat5a* gene was observed.^43^ Notably, several integration sites were shared by different tumors of this strain.^32, 41, 43^ In particular, a 170 bp segment within the second intron was found to be the hotspot for MLV integration (Figure 3). Furthermore, within this region, several nucleotides were shared by different pre-B-cell tumors.

### Hotspots in *Zfp521*

The *Zfp521* (zinc finger protein 521) gene was identified as a common integration site 3 (*Evi3*) in the genomes of B-cell lymphomas in the AKXD mouse strain.^45, 55^ We also reported that this gene is the most frequent integration site as well in pre-B-cell lymphoma in SL/Kh mice.^32^ The encoded ZFP521 regulates and activates pre-B-cell receptor signal pathways.^45^

**Figure 4:**
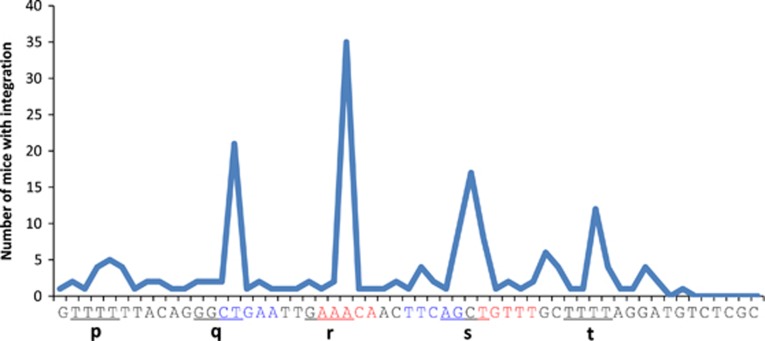
MLV integration within *Zfp521.* The numbers of mice with MLV integration are presented. In the vertical sequence of *Zfp521*, underlines represent the four oligonucleotides duplicated by the integration in the host cell genome when the integration occurs at p, q, r, s, or t. There were mice that have more than one integration sites in the lymphoma tissue genome. The r and s positions are shared by hematopoietic tumor of SL/Kh and AKXD strains. Four integration site data are added to our previously data. A hotspot ‘s' consists of two nucleotide nucleotides G and C.^45, 55^

In more than 140 male SL/Kh mice, >80% pe-B-lymphoma genomes acquired the integrated proviral genome.^32^ Specifically, the integration sites in the genome of pre-B-cell tumors in SL/Kh mice (p, q, r, s, t) are shown in Figure 4. The integration occurred once or twice within a 50 bp segment located in the region of the second to the third exon of *Zfp521* in the pre-B-cell lymphoma genome during the lifetime of the mouse. Two independent integrations into the identical gene in the genome of a single mouse are undoubtedly a rare occurrence. Therefore, we can state that the second to the third exon is one of the hotspots of MLV integration.

### Common features of hotspot sequences

As discussed in above, the sharing at the nucleotide level within the integration tissues in the hematopoietic tumors are not rare events as would be expected, as shown by SL3-3 integration in *c-Myc* and *Emv11* in *Zfp521*, in spite of the fact that it is statistically almost impossible that MLV targets the specified short oligonucleotide as the integration sites. Therefore, the common features of these hotspots within the host genes should be considered. To date, reports have indicated that the promoter region and enhancer comprise the targets of the integration,^9, 10^ or transcriptional active site are favored for intefration.^8, 56^ With respect to the latter and as discussed in further detail later, BET mediates the association of MLV IN and the transcriptional start site in the open chromatin of the host cell.^10^

With respect to DNA structure, the integrations within *Zfp521* frequently occurred at ~10 bp intervals and were symmetrically distributed on either side of the most frequent integration site (p, q, r, s, t in Figure 4).^45, 55^ The 10 bp intervals suggest that MLV integration sites are distributed on the nucleosome surface of the 10 bp periodical outward-facing DNA major grooves in chromatin. To our knowledge, no distinct data has been published regarding 10 bp periodical integration of MLV into the host genome; however, it has been demonstrated that HIV-1 integration sites are periodically distributed on the nucleosome surface by *statistical* analysis.^46^ Our data of 10 bp interval between integration in *Zfp521* provides the first direct evidence of periodical retroviral integration.

The transcriptional factor-binding motifs within the promoter and enhancer might also be involved in the choice of integration site.^57^ In addition, the junction of DNA between the viral and host genome might generate novel motifs required for binding transcription factors and other adaptor proteins, to effect higher transcription of the target gene.^32, 41^ For example, the MLV integration site induces the formation of transcription factor complexes on palindromic sequences during the development of pre-B lymphomagenesis and alters the transcriptional activity of *Stat5a*. Conversely, the transcription factor preference for particular DNA sequence motifs might determine the selection of the integration site. MLV integration induces the formation of transcription factor complexes consisting of GATA, CREB and C/EBPβ on palindromic sequences and on the TATA-box in the *Stat5a* gene. The presumed secondary structure in the open chromatin such as the hairpin structure in the 170 bp segment in *Stat5a* might assist the binding of transcription factors that enhance host gene transcription (Figure 2).^38^ Furthermore, the palindromic motifs might contribute to posttranscriptional stabilization of MLV-host gene chimera RNA through the generation of secondary structure after transcription or of open chromatin structure that controls the transcriptional activity of the host gene when the double helix DNA is rewound. In actuality, such DNA structures are anticipated using *m-fold* analysis (http://mfold.rna.albany.edu/?q=mfold/DNA-Folding-Form) (Figure 3).

Previously, Holman *et al.*^58^ also suggested that palindromes are statistically favored by MLV integration. Palindromic motifs were also observed in the hotspots in *c-Myc*, *Stat5a*, and *Zfp521* In *Zfp521*, the integration occurred at the site indicated as ‘:' in the sequence 5′-CTGAATTG AAAC:AACTTCAGCTGTTT-3′ with 10 bp periodicity at p, q, r, s and t (Figure 4).^45^ Such alternating palindrome sequences are definitely rare motifs. It is noticed that the integration occurs at the middle position, *r*, of the above sequence, suggesting that the sequence may confer the selectivity of the integration into the position surrounding the palindrome. This plot represents a probability density function of MLV insertional mutation. *Pim1* and *Sox4* are also targets of MLV integration in hematopoietic tumor genomes.^12, 27, 41^ In *Pim1* (M13945), the hotspots of integration are positioned in the palindromic sequences at the site indicated by a colon (‘:') as follows: 5′-CCCTGCG:TGAC:GACGCAGGG-3′ and 5′-CCAGGTCCCTGGAGGAGCCTC:CCAC:AAGGGAAAGAGACTACTTCACTGGTCCTGG-3′, where the pair of underlined sequences and dotted-underlined sequences represent palindromic sequences. In *Sox4* (AX695422), the integration sites are positioned near the middle of the palindrome as well in 5′-GGAGCGCGGGGGCGTTAGTGGA:CCCGCG:CT:CC-3′ (Table 1). The preference for the palindrome requires a rigorous statistical study. However, as the sharing of integration sites by different tumors has been found to not be a rare event as had been expected, the DNA structure factor may contribute to the integration site selection.

To better understand the contribution of the host gene to MLV integration, we need to review recent research advances in the control of open chromatin regions in association with IN interaction with histones and its binding to the host gene by recruitment of BET.

## Recent advance of molecular basis of MLV integration

### BET proteins and MLV integration

Recent studies have yielded great advancements in our understanding of MLV integration.^59^ BET proteins (Brd2, Brd3 and Brd4) are the cellular-binding partners of MLV IN and preferentially engage open chromatin regions that are enriched for transcription start sites, CpG islands, DNaseI-hypersensitive sites and proto-oncogenes.^11, 56, 60, 61, 62, 63^ BET family proteins behave as a scaffold on chromatin to recruit E2F proteins, histone deacetylases, histone H4-specific acetyltransferases including GCN5, and chromatin remodeling proteins.^64^ The BET proteins are characterized by an extraterminal domain and two N-terminal bromodomains that recognize and bind acetylated lysines on histone H3 and H4 tails on chromatin.^65^ *In vitro* interaction analysis and co-immunoprecipitation of MLV IN in human cells revealed that different MLV IN domains including the C-terminal domain, the catalytic core domain and the IN C-terminus interacts with the BET family of proteins.^66^ It has also been shown that purified recombinant Brd4 (1–720) bound with high affinity to MLV IN and stimulated MLV concerted integration *in vitro*.^59^ Through the recognition of open chromatin structure, BET proteins have been suggested to contribute to the tethering of the MLV pre-integration complex to the host DNA.^11, 67^ Furthermore, BET protein knockdown and treatment with the cell-permeable small molecule JQ-1, a BET bromodomain inhibitor, were shown to reduce MLV integration frequencies at transcription start sites.^60^ These observations suggest that BET proteins navigate the MLV genome and promote efficient MLV integration around transcription start sites associated with an open chromatin structure.^56^

In addition, BRD4 has a critical role in germinal center response by regulating Bcl-6 and nuclear factor-κB activation.^68^ The double bromodomain protein Brd2 promotes B-cell expansion and mitogenesis.^69^ Accordingly, these proteins might broadly contribute to tumorigenesis in cooperation with MLV integration in the development of spontaneous lymphoma.^70^ Recent advances suggest that integration selection needs to be understood in the context of the epigenetic modification of histones or nucleosomes. Such control mechanisms of transcriptional activity will likely provide novel standpoints of oncogene function and a better understanding of MLV insertional mutagenesis as well.

## Conclusion

Detailed analysis of MLV integration between tumors within and between mouse strains indicates that the convergence of integration sites within a narrow oligonucleotide range is not necessarily a rare event, and that the integration mutagenesis process might be unique among tumor mutagenesis mechanisms. Furthermore, specific DNA structural factors may contribute to the integration site selection, facilitating the generation of ‘hotspot' motifs in tumor genomes.

## Figures and Tables

**Table 1:** Each host gene locus is indicated via its registered number in GenBank

*Host gene*	*Location*	*Previously reported sites*	*SL/Kh*	*Tumor phenotype*	*Reference*
*c-myc*	Promoter				
M12345					
		725		T cell	^47^
		768		T cell	^71^
		773	773	T cell	^72^
		782, 788		Lymphoma	AF524678^73^
		801	804	B cell	AF524315^73^
		811		T cell	^72^
		820		B cell	^47^
		832	832	Unknown	AF524324^73^
			853	Pre-B cell	
		864	864	T cell	Z69825^74^
		876, 886, 889	889	B cell	BH859547^26^
		926		Unknown	AF193142^5^
			932		
		939	939	T, B mixed	^5^
		966	966	B cell	^75^
*Pim-1*	3′ of the last	6980	6980	B cell	^77^
M13945	Eleventh exon	6982		B cell	^77^
		6991		T cell	^78^
		7000, 7005	7005	T cell	^78^
		7064	7063	T cell	^78^
*Sox4*					
AX695422	First exon				
		12 168		B cell, pre-B cell	^26^
		12 173		B cell	^75^
		12 175		B cell	^75^
		12 176		T cell	^26^
		12 182		Pre-B cell myeloid	^26^
*N-myc*	3′	6307		ND	^72^
M12731		6311		T cell	^5^
		6313, 6314, 6315			^76^
		6328	6328	T, B mix	^5^
		6329	6336	Lymphoma	^73^
		6344	6338	Pre-B cell	^50^
		6345	6344	Pre-B cell	^26^
*Zfp521*	First intron				
AC142257		83 098, 83 101	83 098	B cell, pre-B cell	^45, 55^
		83 117	83 117	B cell, pre-B cell	^45, 55^
*Meis 1*					
AL603984	Upstream of the first exon		199 385		
		199 389	199 389	B cell	BH860081^26^
		199 389		Myeloid	BH860521^26^
AL645570	Eleventh intron		77 862	Pre-B cell	
			77 872	Pre-B cell	
		77 878		Myeloid	BH860077^26^
*Cyclin D2*	Promoter				
AC163747		130 896		B cell	^77^
		130 903		T cell	^75^

SL/Kh indicates the integration sites in pre-B-cell lymphoma in SL/Kh mice. The superscripted numbers indicate the reference number in the text. Previous reported sites indicate the nucleotide number based on the GenBank sequence for the individual accession numbers listed. In the *c-myc* gene, No. 1491 represents the transcriptional start nucleotide. ND, no determined.
